# Refractory Immunoglobulin A (IgA) Vasculitis in an Elderly Patient: A Case Report

**DOI:** 10.7759/cureus.28996

**Published:** 2022-09-10

**Authors:** Nozomi Nishikura, Ryuichi Ohta, Noritaka Katagiri, Tatsuhiko Okayasu, Chiaki Sano

**Affiliations:** 1 Community Care, Unnan City Hospital, Unnan, JPN; 2 Communiy Care, Unnan City Hospital, Unnan, JPN; 3 Family Medicine, Shimane University Faculty of Medicine, Izumo, JPN; 4 Family Medicine, International University of Health and Welfare, Tokyo, JPN; 5 Community Medicine Management, Shimane University Faculty of Medicine, Izumo, JPN

**Keywords:** general medicine, rural hospital, older, azathioprine, immunosuppressant, prednisolone, immunoglobulin a vasculitis

## Abstract

Immunoglobulin A (IgA) vasculitis is small-vessel arteritis triggered by autoimmunity and allergies. IgA vasculitis among elderly patients is rare, and there is a lack of evidence regarding the choice of medicine and treatment duration. The main treatment for IgA vasculitis is steroids which can be cured with a small dose of prednisolone without immunosuppressants. Here, we report a case of a 90-year-old patient with the chief complaint of appetite loss and purpura on the legs who was diagnosed with IgA vasculitis based on biopsy results. The patient was initially treated with prednisolone effectively but exacerbated with steroid tapering, eventually requiring the use of an immunosuppressant. This case highlights the importance of monitoring the symptoms of IgA vasculitis while tapering steroids and clarifying the timing of immunosuppressant initiation.

## Introduction

Immunoglobulin A (IgA) vasculitis is a type of small arteritis triggered by autoimmunity and allergies. IgA vasculitis involves the deposition of IgA in the arteries [1]. The typical symptom is palpable purpura on the extremities [2]. It is common in children and rare in the elderly [3]. The symptoms in children may be mild to moderate, with purpura and nephritis [3]. In older patients, however, IgA vasculitis can cause systemic symptoms such as gastrointestinal bleeding and acute renal failure [4,5]. The diagnosis can be made by biopsy of the skin with palpable purpura and detecting leukocytoclastic vasculitis [1]. The trigger for the disease is not clearly known, but viral infections and allergic reactions to drugs and foods are possible.

IgA vasculitis in older patients has been found to be rare, and there is a lack of evidence for treatment. The main treatment for IgA vasculitis is steroids, and most cases can be cured with a small dose of prednisolone without immunosuppressants [6]. The prednisolone dose is then tapered and discontinued. Few patients, however, may require long-term steroid use and immunosuppressants to slow disease progression [1]. We encountered an older patient with chief complaints of fatigue and purpura of the legs. Based on the biopsy results, the patient was diagnosed with IgA vasculitis. The initial reaction to prednisolone was effective, but the patient experienced an exacerbation of the symptoms and eventually required the use of an immunosuppressant. This case highlights the importance of monitoring the symptoms of IgA vasculitis while tapering steroids and clarifying the timing of immunosuppressant initiation.

## Case presentation

A 90-year-old woman was admitted to our hospital complaining of loss of appetite. Twelve days before admission, the patient noticed petechiae in the anterior parts of her lower legs. Eight days before admission, she visited our hospital for an investigation of a rash on her lower leg. Physical examination revealed palpable purpura in the anterior parts of both lower legs. Further, she was consulted by a dermatologist and underwent further investigation using a purpura biopsy. Two days before admission, she experienced nausea and vomiting, causing a loss of appetite. She had a history of repeated hospitalizations due to acute exacerbation of chronic heart failure. At the time of admission, her medical history included dyslipidemia and cataracts. She did not take any medication regularly.

**Figure 1:**
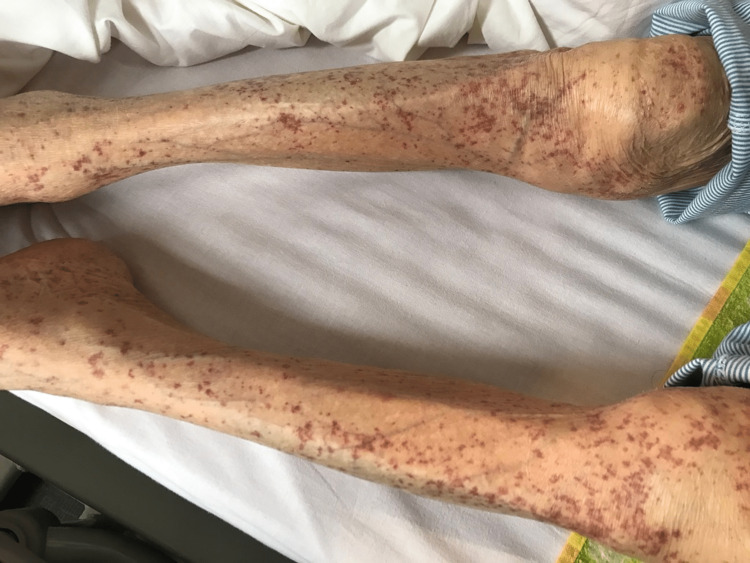
Palpable purpura on the lower extremities

Her vital signs at the time of admission were as follows: blood pressure 137/70 mmHg; pulse rate 83 beats/min; temperature 36.3 °C; respiratory rate 15 breaths/min; and oxygen saturation (SpO2) 98% (room air). Physical examination revealed that the abdomen was distended with tenderness in the lower part and without rebound or percussion tenderness. Palpable purpura was observed on the skin of the abdomen and extremities (Figure 1).

**Figure 2:**
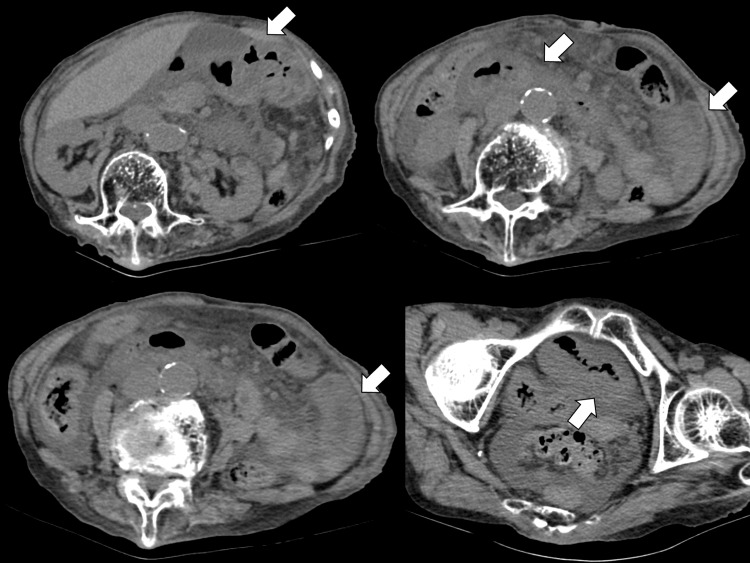
Computed tomography images of the abdomen The computed tomography shows the edematous walls of the stomach, duodenum, and small intestine with skipped lesions (white arrows)

Further, physical examination revealed no abnormalities in the head and chest. Abdominal ultrasonography revealed ascites, edema, and dilatation of the small intestine. To investigate the condition of the abdominal inflammation, contrast-enhanced abdominal computed tomography was performed. Computed tomography (CT) revealed an enhanced small intestine with skipped lesions (Figure 2).

Blood tests showed mild renal damage and inflammation, but no antinuclear antibodies were detected (Table 1). 

**Table 1:** Initial laboratory data for the patient PT, prothrombin time; INR, international normalized ratio; APTT, activated partial thromboplastin time; eGFR, estimated glomerular filtration rate; CK, creatine kinase; CRP, C-reactive protein; TSH, thyroid-stimulating hormone; Ig, immunoglobulin; HCV, hepatitis C virus; SARS-CoV-2, severe acute respiratory syndrome coronavirus 2; HIV, human immunodeficiency virus; HBs, hepatitis B surface antigen; HBc, hepatitis B core antigen; PR3, proteinase 3; MPO, myeloperoxidase; ANCA, anti-neutrophil cytoplasmic antibody

Marker	Observed Levels	Reference Values
White blood cells	7.90	3.5–9.1 × 10^3^/μL
Neutrophils	85.9	44.0–72.0%
Lymphocytes	10.3	18.0–59.0%
Monocytes	3.4	0.0–12.0%
Eosinophils	0.0	0.0–10.0%
Basophils	0.4	0.0–3.0%
Red blood cells	3.27	3.76–5.50 × 10^6^/μL
Hemoglobin	11.1	11.3–15.2 g/dL
Hematocrit	32.4	33.4–44.9%
Mean corpuscular volume	99.0	79.0–100.0 fl
Platelets	30.2	13.0–36.9 × 10^4^/μL
PT-INR	0.91	<1.50
APTT	26.6	25–40 seconds
Fibrinogen	326.8	200–400 mg/dL
Fibrinogen degradation products	50.0	<5 μg/mL
Erythrocyte sedimentation rate	38	2–10 mm/hour
Total protein	6.4	6.5–8.3 g/dL
Albumin	3.6	3.8–5.3 g/dL
Total bilirubin	0.6	0.2–1.2 mg/dL
Aspartate aminotransferase	23	8–38 IU/L
Alanine aminotransferase	12	4–43 IU/L
Alkaline phosphatase	51	106–322 U/L
γ-Glutamyl transpeptidase	8	<48 IU/L
Lactate dehydrogenase	210	121–245 U/L
Blood urea nitrogen	26.9	8–20 mg/dL
Creatinine	0.68	0.40–1.10 mg/dL
eGFR	60.0	>60.0 mL/min/L
Serum Sodium	136	135–150 mEq/L
Serum Potassium	5.1	3.5–5.3 mEq/L
Serum Chloride	100	98–110 mEq/L
CK	178	56–244 U/L
CRP	2.00	<0.30 mg/dL
TSH	2.21	0.35–4.94 μIU/mL
Free T4	1.1	0.70–1.48 ng/dL
IgG	1461	870–1700 mg/dL
IgM	52	35–220 mg/dL
IgA	237	110–410 mg/dL
IgE	123	<173 mg/dL
HBs antigen	0.0	0.00-0.04 U/mL
HBs antibody	0.0	mIU/mL
HCV antibody	0.00	0.00-0.99S/CO
Syphilis treponema antibody	0.00	S/CO
SARS-CoV-2 antigen	negative	
C3	95	86-160 mg/dL
C4	33	17-45 mg/dL
Anti-nuclear antibodies	<40	<40
PR3-ANCA	<1.0	<3.5 U/mL
MPO-ANCA	<1.0	<3.5 U/mL
Urine test		
Leukocyte	negative	
Nitrite	negative	
Protein	(+)	
Glucose	negative	
Urobilinogen	normal	
Bilirubin	negative	
Ketone	(2+)	
Blood	(+)	
pH	5.0	
Specific gravity	1.025	
Fecal occult blood	negative	

**Figure 3:**
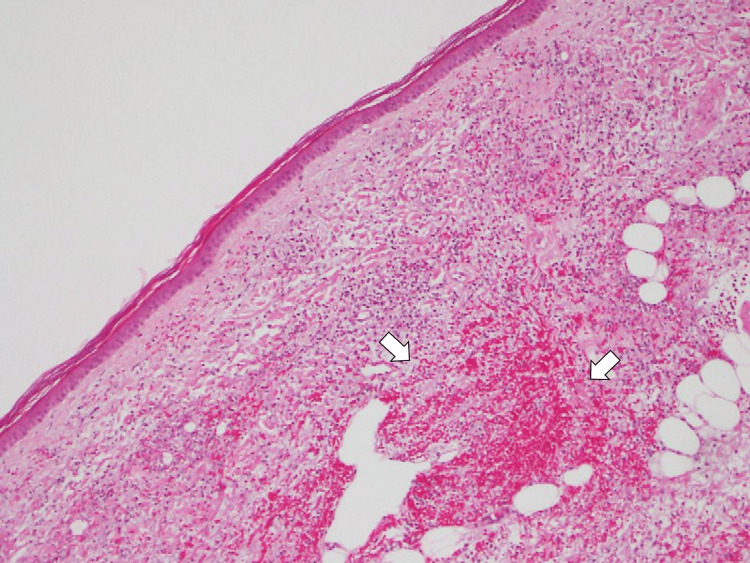
Pathological investigation image The pathological investigation of the purpura of the lower legs shows leukocytoclastic vasculitis (white arrows)

Pathological examination of purpura on the day of hospitalization revealed leukocytoclastic vasculitis in the arteries of the skin (Figure 3).

Accordingly, we diagnosed IgA vasculitis and initiated prednisolone at a dose of 20 mg/day. On day 17 of hospitalization, the patient’s symptoms were relieved, and the purpura disappeared. Prednisolone was gradually tapered to 5 mg over two weeks. Because of disuse syndrome, the patient was transferred to the rehabilitation unit on day 52. 

On day 15 of the rehabilitation unit, the prednisolone dose was tapered to 3 mg/day. Five days later, the purpura appeared on the lower legs again, and the patient developed bloody stool with a fever of 37.3 °C. We considered the exacerbation of IgA vasculitis, increased the prednisolone dose to 5 mg, and started azathioprine at 25 mg based on the previous reports with the effectiveness and safety panel among older patients [6]. Accordingly, her fever and blood stools resolved, and her activities of daily living recovered in the rehabilitation unit. On day 77 of hospitalization, she was discharged to her home. Fourteen days after symptom exacerbation, the azathioprine dose was increased to 50 mg, and the prednisolone dose was tapered again without relapse. 

## Discussion

This case report describes an elderly patient with anorexia followed by the appearance of purpura, which led to a diagnosis of IgA vasculitis based on a biopsy indicative of leukocytoclastic vasculitis. Steroid therapy was effective initially; however, there was a need to add an immunosuppressant to taper the steroid effectively. This case presents a quick diagnosis of IgA vasculitis and a concrete treatment strategy for the refractory clinical course of the disease.

The detailed mechanism of IgA vasculitis is unknown and may be a type 3 allergic reaction in which neutrophils damage the vessel wall by forming immune complexes and initiating an inflammatory reaction. IgA vasculitis is rare in the elderly, but it is accompanied by a higher risk of a severe clinical course due to a higher frequency of multiorgan failure than in children [7-10]. Although the disease often resolves spontaneously, it is treated with steroids and immunosuppressive agents when symptoms are severe or when the disease is refractory. Because this case's clinical course was refractory to steroids, azathioprine was used as an immunosuppressant.

Steroids are expected to improve abdominal pain associated with IgA vasculitis but do not necessarily shorten the clinical course of the disease; therefore, steroids should be used in the short term to prevent various complications [11,12]. Spontaneous remission usually occurs within a few weeks. However, immunosuppressive therapy may also treat refractory IgA vasculitis [2]. However, steroid use in the elderly is associated with fatal side effects such as increased susceptibility to infection and femoral neck fractures [6]. Reduced renal function in the elderly may result in higher blood concentrations of steroids, and prolonged steroid therapy is critical for immune suppression [9,13]. Steroids suppress inflammatory cytokines and prevent neutrophil migration. Therefore, steroid therapy for IgA vasculitis in elderly patients should be short and be administered at low doses. We tapered the prednisolone dose as quickly as possible in this case by following the patient’s symptoms. Accordingly, the concomitant use of immunosuppressive agents should be considered if the patient is refractory to steroids or if their symptoms flare up after tapering.

One useful immunosuppressant for IgA vasculitis is azathioprine, which should be used in combination with prednisolone in elderly patients. Azathioprine inhibits leukocyte proliferation by blocking purine synthesis [2,14,15]. Different mechanisms may contribute to remission and shortening IgA vasculitis time. Since azathioprine is used to treat Crohn's disease and ulcerative colitis, which are caused by B-cell abnormalities, it is suggested to be more effective than other immunosuppressants for IgA vasculitis with abdominal manifestations [2,14,15]. Although evidence in the elderly has not been established, studies in children have shown an increased remission rate with the combination of azathioprine and corticosteroids [3]. Case reports of IgA vasculitis in the elderly have reported comparable results [2,14,15]. In this case, azathioprine effectively suppresses the symptoms and tapers the prednisolone dose. Azathioprine has been approved by safety panels for use in elderly patients. Accordingly, azathioprine is important for the effective treatment of IgA vasculitis in elderly patients.

Treating elderly patients diagnosed with IgA vasculitis can be challenging, and general physicians should precisely monitor their symptoms to control medications. Elderly patients with various diseases may exhibit different symptoms compared to symptoms in younger patients [16,17]. Trivial symptoms can be linked to critical conditions, such as infarction and infection [18,19]. Physicians treating IgA vasculitis in elderly patients must carefully monitor the symptoms of IgA vasculitis, such as abdominal and skin symptoms, steroid side effects, and systemic conditions, including renal function. Additionally, the treatment should be short and administered at low doses to reduce the side effects of steroids. Immunosuppressive therapy should be considered in treating IgA vasculitis in elderly patients.

## Conclusions

This case shows the difficulty of treating IgA vasculitis with steroids and tapering the treatment in elderly patients. Azathioprine might be an effective alternative to steroids and can be safely used even for vasculitis in older patients. General physicians should monitor the symptoms of IgA vasculitis precisely and use steroids and immunosuppressants based on patients’ symptoms.
